# Effects of Low-Dose Non-Caloric Sweetener Consumption on Gut Microbiota in Mice

**DOI:** 10.3390/nu9060560

**Published:** 2017-06-01

**Authors:** Takashi Uebanso, Ai Ohnishi, Reiko Kitayama, Ayumi Yoshimoto, Mutsumi Nakahashi, Takaaki Shimohata, Kazuaki Mawatari, Akira Takahashi

**Affiliations:** 1 Department of Preventive Environment and Nutrition, Institute of Biomedical Sciences, Tokushima University Graduate School, 3-18-15, Kuramoto, Tokushima 770-8503, Japan; fqwbo@yahoo.co.jp (A.O.); c201202050@tokushima-u.ac.jp (R.K.); sea.by15.koko@gmail.com (A.Y.); shimohata@tokushima-u.ac.jp (T.S.); mawatari@tokushima-u.ac.jp (K.M.); akiratak@tokushima-u.ac.jp (A.T.); 2 Graduate School of Bioscience and Bioindustry, Tokushima University, Tokushima 770-8503, Japan; nakahashim@tokushima-u.ac.jp

**Keywords:** sucralose, acesulfame-K, gut microbiota, cholesterol, bile acid, acceptable daily intake, DGGE, CE-MS, artificial sweetener

## Abstract

Non-caloric artificial sweeteners (NASs) provide sweet tastes to food without adding calories or glucose. NASs can be used as alternative sweeteners for controlling blood glucose levels and weight gain. Although the consumption of NASs has increased over the past decade in Japan and other countries, whether these sweeteners affect the composition of the gut microbiome is unclear. In the present study, we examined the effects of sucralose or acesulfame-K ingestion (at most the maximum acceptable daily intake (ADI) levels, 15 mg/kg body weight) on the gut microbiome in mice. Consumption of sucralose, but not acesulfame-K, for 8 weeks reduced the relative amount of *Clostridium cluster XIVa* in feces. Meanwhile, sucralose and acesulfame-K did not increase food intake, body weight gain or liver weight, or fat in the epididymis or cecum. Only sucralose intake increased the concentration of hepatic cholesterol and cholic acid. Moreover, the relative concentration of butyrate and the ratio of secondary/primary bile acids in luminal metabolites increased with sucralose consumption in a dose-dependent manner. These results suggest that daily intake of maximum ADI levels of sucralose, but not acesulfame-K, affected the relative amount of the *Clostridium cluster XIVa* in fecal microbiome and cholesterol bile acid metabolism in mice.

## 1. Introduction

Unlike sugars, non-caloric artificial sweeteners (NASs), including saccharin, sucralose, acesulfame-K, and aspartame, provide sweet taste to food without adding energy content [1]. Because NASs do not add energy content or glucose, they can be used as an alternative sugar source for controlling blood glucose levels and body weight in diabetic patients [2]. More recently, the use of NASs by the general public has become a popular approach to reduce energy intake, glycemic load and weight gain. Consumption of NASs has increased in the past decade in Japan, the United States, and other countries [3,4]. Reports from the Japan National Health and Nutrition Survey showed that between 2002 and 2015 consumption of sucralose and acesulfame-K increased from 0.310 mg/kg body weight/day to 0.825 mg/kg body weight/day (sucralose) and from 0.736 mg/kg body weight/day to 1.357 mg/kg body weight/day (acesulfame-K) [4]. In contrast, between 2002 and 2015, the consumption of other NASs, including aspartame (from 5.853 to 0 mg/kg body weight/day) and saccharin (from 0.648 to 0.112 mg/kg body weight/day), declined in Japan [4]. Thus, studies of the effects of NASs, particularly sucralose and acesulfame-K, on metabolism are important to provide guidance on the amount of NASs that can be consumed. 

In the past decade, several studies examined the effects of NASs on appetite, weight gain, incidence of obesity, and metabolic disorders, but the results were not consistent [3,5]. In a systematic review, Imamura et al. found that individuals who consumed higher amounts of beverages made with artificial sweeteners had an 8% increased incidence of type 2 diabetes, even after adjustment for adiposity [6], whereas Kim et al. and Cheungpasitporn et al. showed that these beverages were associated with an increased risk of hypertension [7,8]. Meanwhile, Cheungpasitporn et al. reported that the pooled risk ratios (RR) of five study samples of chronic kidney disease in patients who consumed artificially sweetened soda were not statistically significant (RR: 1.33, 95% confidence intervals 0.82–2.15) [9], although it should be noted that neither the Cheungpasitporn et al. study nor the Kim et al. study made adjustments for adiposity.

Recently, a study by Suez J et al. suggested that saccharin, aspartame, or sucralose consumption could induce glucose intolerance by promoting gut dysbiosis [10]. Sucralose is rarely absorbed in the mammalian gut, but this NAS can affect non-mammalian cells, including gut bacteria [11]. Sucralose has been shown to increase serotonin, which initiates peristaltic and secretory activity [12,13]. In rats, consumption of sweeteners containing sucralose (e.g., Splenda) reduced the numbers of intestinal bacteria [14]. In addition, chronic consumption of sucralose triggered increased food intake in mice and flies [15]. However, because this study used high amounts of NASs, particularly sucralose (approximately 20-fold higher than the acceptable daily intake (ADI) [16]), whether lower doses of sucralose or acesulfame-K (at most the maximum ADI levels of 15 mg/kg body weight) also affect the gut microbiome and food intake are unclear. In the present study, we examined the effect of low-dose pure sucralose or acesulfame-K consumption on the gut microbiome and host metabolism in mice.

## 2. Materials and Methods

### 2.1. Animals

Male and female C57Bl/6J mice (8 weeks old) were purchased from a local breeding colony (Charles River Japan, Yokohama, Japan). After acclimatization for several weeks, male and female mice were mated, and the resulting male pups were used in this study, including in Experiment 1 and 2. Mice were housed in cages maintained at constant temperature (23 °C ± 2 °C) and humidity (65–75%) with a 12 h light (8:00–20:00)/12 h dark cycle. Prior to the initiation of our study, the mice were allowed free access to standard rodent diet (MF; Oriental Yeast) and water. 

### 2.2. Experimental Design

In all experiments, mice were allowed free access to standard rodent diet (AIN93G; Oriental Yeast). In Experiment 1, 4-week-old male mice were divided into 3 groups and treated for 8 weeks as follows: Control group mice were given distilled water (C, *n* = 8); low-dose sucralose (LS) group mice were given a sucralose solution of 1.5 mg/kg body weight per day (*n* = 8); and high dose sucralose (HS) group mice were given sucralose solution of 15 mg/kg body weight per day, which is equal to the maximum ADI (*n* = 8) [16]. In Experiment 2, 4-week-old male mice were divided into 2 groups and treated for 8 weeks as follows: Control group mice were given distilled water (C, *n* = 8); and acesulfame-K (AK) group mice were given an acesulfame-K solution of 15 mg/kg body weight per day, which is equal to the ADI (*n* = 9) [16]. For both experiments, body weight and fluid intake were measured 3 times a week. We used pair-feeding like a method to manage the sweetener consumption of mice. The sweetener concentration was calculated from their fluid intake/day and body weight. Then, we adjusted sucralose concentration of drinking water every day or every two days to regulate sucralose consumption. All mice were euthanized at noon on the final day of the experiment and the blood, cecum, cecal contents, feces, and liver tissue were collected. The University of Tokushima Animal Use Committee approved the study (T14010), and mice were maintained according to the National Institutes of Health Guidelines for the Care and Use of Laboratory Animals.

### 2.3. Plasma and Hepatic Lipid Concentrations

Hepatic lipids were extracted and measured as previously described [14]. Plasma and liver TG and total cholesterol concentrations were measured using Triglyceride-E and Cholesterol-E tests (Wako Pure Chemical Industries, Osaka, Japan), respectively.

### 2.4. RNA Preparation and Quantitative Reverse Transcriptase Polymerase Chain Reaction

Extraction of total RNA, cDNA synthesis, and real-time polymerase chain reaction (PCR) analyses were performed as described previously [17]. The relative abundance of each target transcript was calculated by normalizing the amount of amplified product to the amount of constitutively expressed β-actin mRNA. The primer sequences are listed in Table S1.

### 2.5. Extraction of Genomic DNA and Quantitative Polymerase Chain Reaction

Genomic DNA from fecal and cecal content samples was isolated using a Favorprep Stool DNA Isolation Mini Kit (FAVORGEN Biotech Corp., Ping-Tung, Taiwan) according to the manufacturer’s protocol. The relative abundance of each target bacterial 16S rRNA gene copy (primer sequences are shown in Supplemental Table S1) was calculated by normalizing relative to the amount of amplified product from all bacteria 16S rRNA gene copies.

### 2.6. PCR Denaturing Gradient Gel Electrophoresis (DGGE) Analysis

Denaturing gradient gel electrophoresis (DGGE) was carried out as previously reported [18] using a DCode^TM^ Universal Mutation Detection System instrument and a Model 475 gradient former according to the manufacturer’s instructions (Bio-Rad Labs, Hercules, CA, USA). The V2-V3 region of the 16S rRNA genes (positions 339 to 539 in the *Escherichia coli* gene) of bacteria in the gut samples was amplified by primers HDA1-GC and HDA2 as described by Walter et al. [19]. PCR reaction mixtures and the amplification program were the same as described previously [19] except that 30 amplification cycles were used. The denaturing gradient was formed using two 8% acrylamide gels (acrylamide-bis 37.5:1) with denaturing gradients ranging from 30 to 70% for analysis of the amplified 16S rRNA fragments. The 100% denaturant solution contained 40% (*v*/*v*) deionized formamide, and 7 M urea. PCR products (40 μL) were mixed with 40 μL of loading dye before loading. Gels were run in 0.5 × TAE at 60 °C for 5.2 h at 180 V, 210 mA, stained with Gel Star (Lonza, ME, USA) for 30 min, and analyzed by Chemi Doc MP (Bio-Rad Laboratories, CA, USA). Image Lab software version 5.0 (Bio-Rad) was used for the identification of bands and normalization of band patterns from DGGE gels.

### 2.7. Extraction of Bile Acids

Bile acids were extracted according to Hagio’s method [20]. Stored liver samples and cecal contents were freeze-dried for storage. For analysis, 1 mL of ethanol with nordeoxycholic acid (NDCA) (50 nmol) as an internal standard was added to 50 mg of the freeze-dried samples, which were then subjected to sonication followed by heating at 60 °C for 30 min. After cooling to room temperature, the samples were heated at 100 °C for 3 min and centrifuged at 1600 *g* for 10 min at 15 °C. The supernatants were then collected. Ethanol (1 mL) was added to precipitates and mixed vigorously by vortexing for 1 min before centrifugation at 11,200 *g* for 1 min. The supernatants were collected, pooled, and the liquid was evaporated from the pooled extracts. The extract residue was resuspended in methanol for purification and analysis by liquid chromatography mass spectrometry (LC-MS).

### 2.8. Analysis of Bile Acid Composition

Liquid chromatography (LC) separation was performed using an Agilent LC system (Agilent Technology, Santa Clara, CA, USA) with a gradient elution from a D.N.72770-902 C18 column (1.8 μm, 50 mm × 2.1 mm, D.N.72770-902) at 40 °C and a flow rate of 200 μL/min. The auto sampler was kept at 15 °C. The sample injection volume was 5 μL. Solvent A and Solvent B were 20:80 and 80:20 mixtures, respectively, of acetonitrile–water and each contained 10 mM ammonium acetate. The gradient program was as follows: 0 min—B 5%, 5 min—B 5%, 15 min—B 15%, 20 min—B 25%, 22 min—B 75%, 24 min—B 75%, 27 min—B 5%, post-time 5 min. Specific bile acid peak retention time and *m/z* were confirmed against standards. The Qualitative Analysis feature of MassHunter Acquisition software, version B.06.00 (Agilent), was used to calculate detected peak areas. Data were adjusted according to NDCA peak (internal control) and sample weight.

### 2.9. Metabolome Analysis of Cecum Luminal Contents by Capillary Electrophoresis Electrospray Ionization Time-of-Flight Mass Spectrometry

The cecum luminal contents were immediately frozen in liquid nitrogen and stored at −80 °C until metabolite extraction. Sample tissues were weighed and completely homogenized in 0.5 mL of ice-cold methanol containing 50 μM methionine sulfone and camphor-10-sulfonic acid as internal standards. The homogenates were mixed with 0.5 mL of chloroform and 0.2 mL of ice-cold Milli-Q water. After centrifugation at 2300× *g* for 5 min, the supernatant was centrifugally filtrated through 5 kDa cut-off filters (Millipore, Bedford, MA, USA) at 9100× *g* for 4 to 5 h to remove proteins. The filtrate was then centrifugally concentrated in a vacuum evaporator, dissolved in Milli-Q water, and analyzed by capillary electrophoresis electrospray ionization time-of-flight mass spectrometry (CE-TOFMS) using an Agilent CE system combined with a TOFMS (Agilent Technologies, Palo Alto, CA, USA) as reported by Human Metabolome Technologies, Inc. (HMT) (HMT, Tsuruoka, Japan) [21,22]. Each metabolite was identified and quantified based on the peak information, including *m/z*, migration time, and peak area.

### 2.10. Statistical Analyses

All values are expressed as mean ± S.E. The significance of differences between two groups was assessed using an unpaired two-tailed *t* test. For comparison between more than two groups, analysis of variance (ANOVA) or the Kruskal–Wallis test was used. When a significant difference was found by ANOVA or the Kruskal–Wallis test, post hoc analyses were performed using the Tukey–Kramer protected least significant difference test. Concentration-dependent effects were identified using regression analysis. Spearman’s rank correlation coefficient was used to calculate correlation coefficients between selected variables. Differences were considered significant at *p* < 0.05. Statistical analyses were performed using Mass Profiler Professional (MPP) and Excel–Toukei 2006 (SSRI).

## 3. Results

**Figure 1:**
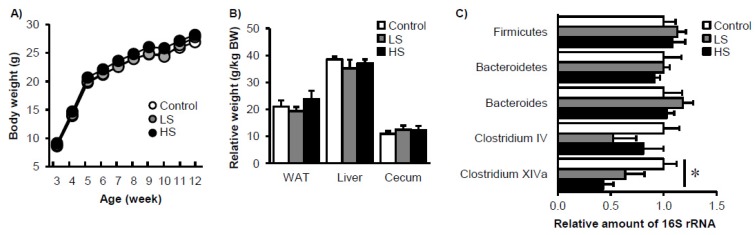
Gradual changes in body composition and gut microbiota in mice fed sucralose. Changes in body weight (**A**), organ weight (**B**), and abundance of specific bacterial phylum and genus (**C**) after 8 weeks of sucralose feeding. Data represent mean ± S.E. (*n* = 8). * Concentration-dependent effects were observed by regression analysis, *p* < 0.05. LS: low sucralose (1.5 mg/kg BW/day), HS: high sucralose (15 mg/kg BW/day).

To elucidate the effect of low-dose sucralose consumption on gut microbiota and host metabolism in mice, either 1.5 mg/kg BW/day (LS) or 15 mg/kg BW/day (HS) was given to mice from weaning in Experiment 1. The actual sucralose intake during Experiment 1 was 1.4 ± 0.1 mg/kg BW/day and 14.2 ± 2.2 mg/kg BW/day in the LS and HS groups, respectively. Water and energy consumption across Experiment 1 was 3.4 ± 0.6 mL/day and 11.0 ± 0.5 kcal/day in the control group, 3.4 ± 0.5 mL/day and 10.9 ± 0.3 kcal/day in the LS group, and 3.7 ± 0.5 mL/day, and 11.1 ± 0.4 kcal/day in the HS group, respectively (*p* > 0.05). During the 8-week duration of Experiment 1, body weight, relative epididymal fat weight, relative liver weight, and relative cecum weight were similar among the three groups (Figure 1A,B). The relative amounts of fecal total bacteria, as well as *Firmicutes* and *Bacteroidetes* phylum bacteria, were also similar among the groups. However, the relative amount of *Clostridium cluster XIVa* was decreased in the presence of sucralose consumption in a dose-dependent manner (Figure 1C). To explore in detail the microbiome composition of the three experimental groups, we next carried out a DGGE analysis. The relative band intensity of different 16S rRNAs from the V2 to V3 region did not differ among the three groups (Figure S1). Furthermore, principal component analysis (PCA) and hierarchical clustering analysis could not distinguish group-dependent changes in the gut microbiome among the three groups (Figure S1; some data not shown). Taken together, low-dose (at most the maximum ADI level) sucralose intake continuously for 8 weeks from weaning reduced the relative amount of *Clostridium cluster XIVa* in feces in mice, but other factors were unaffected.

**Figure 2:**
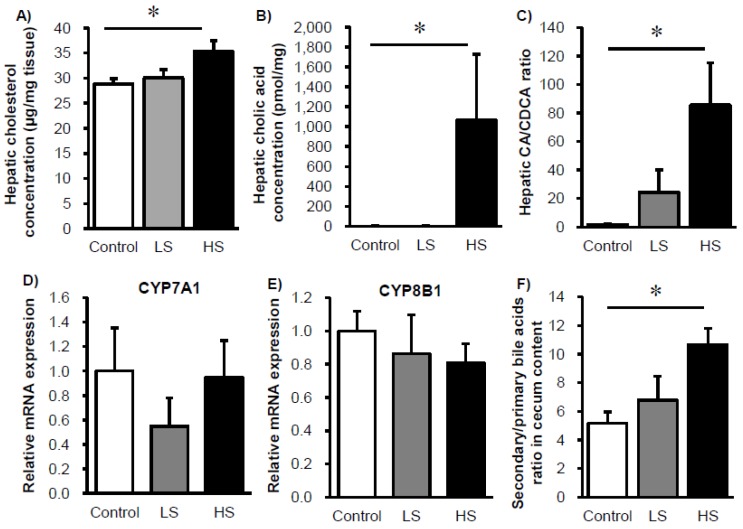
Cholesterol and bile acid metabolism are altered in mice fed sucralose. Changes in hepatic cholesterol (**A**), cholic acid (**B**) CA/CDCA ratio (**C**), mRNA expression levels of CYP7a1 (**D**), CYP8b1 (**E**), and luminal primary and secondary bile acid ratio (**F**) in mice fed sucralose for 8 weeks. Data represent mean ± S.E. (*n* = 8). * Concentration-dependent effects were observed by regression analysis, *p* < 0.05. LS: low sucralose (1.5 mg/kg BW/day), HS: high sucralose (15 mg/kg BW/day).

Given the sucralose-dependent changes in the amount of *Clostridium cluster XIVa* bacteria that affect cholesterol-bile acid metabolism [23], we examined how changes in the gut microbiome affected host metabolism. The hepatic cholesterol concentration was slightly higher in the HS group relative to the control group (Figure 2A). Meanwhile, for bile acids, the cholic acid (CA) concentration and the CA/chenodeoxycholic acid (CDCA) ratio increased with sucralose consumption in a dose-dependent manner (Figure 2B,C). We did not observe any changes in the mRNA expression levels of hepatic cholesterol 7α-hydroxylase (CYP7A1) and sterol 12α-hydroxylase (CYP8B1), which regulate primary bile acid synthesis and the ratio of CA/CDCA, respectively (Figure 2D,E) [24]. On the other hand, in the cecal contents the ratio of secondary bile acids (dehydro-cholic acids (DCA) and lithocholic acid (LCA)) to primary bile acids (CA and CDCA) was increased upon sucralose consumption in a dose-dependent manner (Figure 2F).

**Figure 3:**
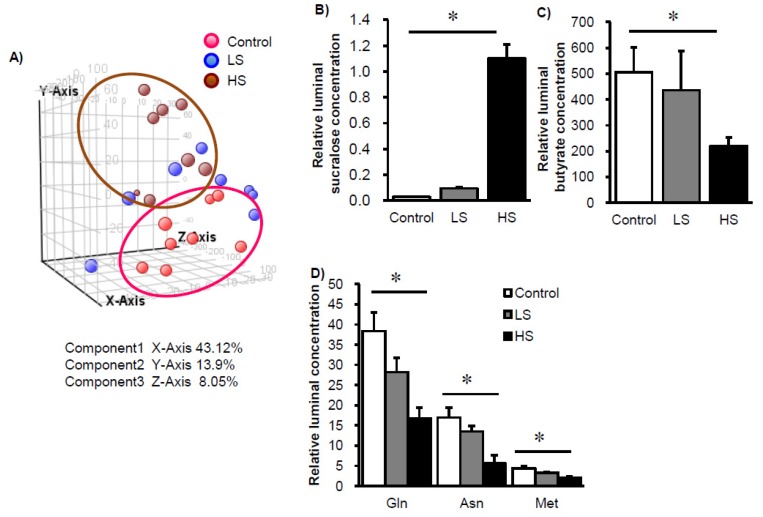
Changes in luminal metabolism in mice fed sucralose. Principle component analysis of 82 luminal metabolites in mice fed sucralose (**A**). Changes in relative concentration of sucralose (**B**), butyrate (**C**), and specific amino acids (**D**) in mice fed sucralose for 8 weeks. Data represent mean ± S.E. (*n* = 8). * Concentration-dependent effects were observed by regression analysis, *p* < 0.05. LS: low sucralose (1.5 mg/kg BW/day), HS: high sucralose (15 mg/kg BW/day).

To further investigate the effects of sucralose intake on luminal metabolites, we conducted CE-MS analysis. In addition to sucralose, we could define 82 metabolites from an HMT metabolite list. From the PCA plot, metabolite patterns for the control group and HS group clearly differed (Figure 3A), and in particular 5 metabolites significantly differed among the three groups (Figure 3B–D). We noted that the butyrate concentration in the cecal contents was reduced in a sucralose dose-dependent manner (Figure 3C), which may be because *Clostridium cluster XIVa* bacteria produce butyrate. These results suggest that low-dose sucralose intake may affect luminal bacterial metabolism. 

**Figure 4:**
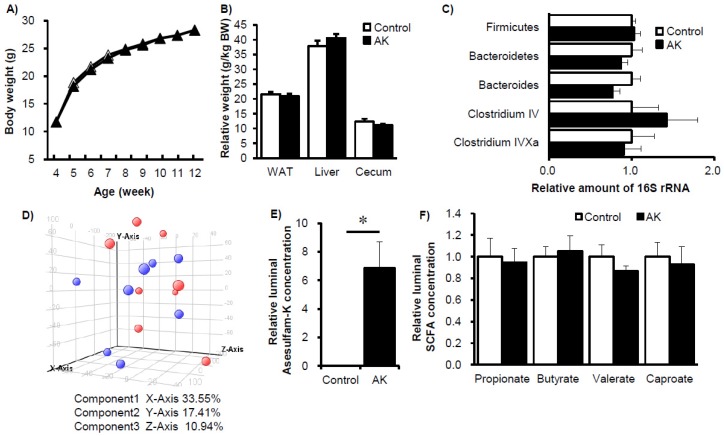
Gradual changes in body composition, gut microbiota and luminal metabolites in mice fed acesulfame-K. Changes in body weight (**A**), organ weight (**B**), and abundance of specific bacterial phylum and genus (**C**) after 8 weeks of acesulfame-K feeding (AK). Principle component analysis of 82 luminal metabolites in acesulfame-K fed mice (**D**). Changes in relative concentration of acesulfame-K (**E**) and short chain fatty acids (**F**) in mice fed acesulfame-K for 8 weeks. Data represent mean ± S.E. (*n* = 8). * *p* < 0.05 as compared to the control group.

To elucidate how acesulfame-K consumed at ADI levels affects the gut microbiome and host metabolism in mice, in Experiment 2 mice were given either distilled water alone (C) or 15 mg/kg BW/day acesulfame-K (AK). The actual acesulfame-K intake during Experiment 2 was 12.9 ± 1.4 mg/kg BW/day for the AK group. Water and energy consumption across Experiment 2 was 3.4 ± 0.2 mL/day and 10.5 ± 0.5 kcal/day in the control group and 3.3 ± 0.3 mL/day and 10.4 ± 0.5 kcal/day in AK group, respectively (*p* > 0.05). During the 8-week duration of Experiment 2, except for the relative liver weight, the body weight, relative epididymal fat weight, and relative cecum weight did not differ between the two groups (Figure 4A,B). In the feces of these mice, the relative amounts of total bacteria, *Firmicutes* phylum, *Bacteroidetes* phylum, and several other genuses were similar between the two groups (Figure 4C). We next carried out a DGGE analysis to explore in greater detail the gut microbiome composition. The relative band intensity of different 16S rRNAs from the V2 to V3 region did not differ significantly between the two groups for either cecal content or feces (Supplemental Figure S2). Principal component analysis (PCA) and hierarchical clustering analysis did not identify any difference between the control and AK groups (Figure S2; some data not shown). Furthermore, luminal metabolome analysis could not distinguish between the two groups, with the exception of the presence of acesulfame-K (Figure 4D–F). Finally, we compared both the control groups from Experiment 1 and Experiment 2 to establish overall reproducibility between independent trials. There are no major differences in the luminal metabolites, which reflect luminal bacterial metabolism activity between the control groups from the both experiments (Figure S3). Taken together, relative to sucralose, low-dose (at most the maximum ADI level) acesulfame-K intake had fewer effects on gut microbiota and its metabolism in mice. 

## 4. Discussion and Conclusions

We found that administration of pure sucralose (14.2 mg/kg BW/day), but not acesulfame-K (12.9 mg/kg BW/day), to mice for 8 weeks slightly but significantly altered gut microbiota. For mice given sucralose, the abundance of *Clostridium XIVa* in feces decreased, although the body weight did not change. Schiffman reported that administration of 100 to 1000 mg/kg BW/day of Splenda (containing 1% w/w sucralose, approximately 1.1 to 11 mg/kg BW/day) for 12 weeks significantly altered the culturable gut microbiota composition and increased body weight in rats [14]. Another report revealed that administration of 10% Sucralite solution (containing 5% sucralose, approximately 333 mg/kg BW/day) for 11 weeks impaired the gut microbiome as evaluated by 16s rRNAs sequencing and glucose tolerance in mice. The differences among the three studies may arise from differences in model species, sucralose purity and dose, diet, and methods used to characterize gut microbiota. In addition to these differences, we but not others started to administrate sweetener from early life of age. Because environmental factors, such as low-dose antibiotics, from early stage of life more significantly affected the gut microbiota [25,26], the age of initiating the sucralose intake may influence the effect of sucralose on gut microbiota. Here, we could define both sucralose and acesulfame-K in luminal metabolites, indicating that the NASs could indeed reach the gut microbial community as reported previously [11]. In the present study, administration of sucralose did not show signs of gastrointestinal tract disturbance, such as peri-anal soiling, scouring, and cecal enlargement as reported in rabbit fed 750 mg/kg/day sucralose [27].

Comparison of bile acid composition in germ-free mice and conventionally raised mice clearly showed that most bile acids are metabolites of the gut microbiome [28]. We found that daily sucralose intake increased the ratio of luminal secondary bile acids to primary bile acids. This increase could not be explained by the reduction in the relative abundance of *Clostridium XIVa* genus bacteria in feces because *Clostridium* genus *cluster XI* and *XIVa* participate in the conversion of primary bile acids to secondary bile acids [29]. We should consider the gut microbiome and host enterohepatic circulation as a complex system in cholesterol–bile acid metabolism and that these processes involve not only conversion and deconjugation of bile acids but also the reabsorption of bile acids. Indeed, the hepatic CA concentration and CA/CDCA ratio were increased by sucralose intake without any changes in the mRNA expression levels of bile acid composition regulators. Among the changes in cholesterol bile acid metabolites observed in this study, CA promotes cholesterol absorption, whereas CDCA does not [30]. This increase in the CA/CDCA ratio may potentially enhance cholesterol absorption, leading to a significant accumulation of cholesterol in the liver, which in turn could promote hypercholesterolemia. Our novel data showed that sucralose intake increases the CA/CDCA ratio, which may in part explain the effect of sucralose intake in regulating cholesterol and bile acid metabolism in vivo.

In contrast to bile acid metabolism, the luminal butyrate concentration decreased with the reduction of the butyrate-producing bacteria of genus *Clostridium XIVa*. Butyrate and other short chain fatty acids have been shown to be effective in reducing colitis through the induction of regulatory T cells [31,32]. One notable result we obtained was that the effects of sucralose feeding on gut microbiota and its metabolism were dose-dependent. These results suggest that sucralose, but not acesulfame-K, could affect luminal bacterial metabolism.

In a recent study in mice, Wang et al. showed that daily intake of jelly containing 7.5 mg sucralose (approximately 2250 mg/kg BW/day for mice weighing 30 g) promoted significant increases in food intake [15]. This increase in a robust appetite-stimulating response to sucralose was confirmed in both tetracycline-treated and germ-free flies, suggesting that this effect is independent of the gut microbiome. In our study, 14.2 mg/kg BW/day sucralose intake did not increase food intake or body weight. Because food intake in rodents is difficult to measure accurately, another well controlled study will help to understand the effect of sucralose on hunger or food intake. In conclusion, we found that consumption of low-dose sucralose, but not acesulfame-K, affected the relative amount of the *Clostridium cluster XIVa* in fecal microbiome and cholesterol bile acid metabolism in mice, even at levels within the ADI. The effect of NASs on gut microbiota and lipid metabolism thus appears to vary according to type and dose of NASs.

## Supplementary Materials

The following are available online at www.mdpi.com/2072-6643/9/6/560/s1; Figure S1: DGGE analysis of 16S V2-V3 rRNA in feces from mice fed sucralose. Band patterns (**A**) and hierarchical clustering of band patterns (**B**) for 16S V2-V3 rRNA in feces from mice fed sucralose for 8 weeks. LS: low sucralose (1.5 mg/kg BW/day), HS: high sucralose (15 mg/kg BW/day), Figure S2: DGGE analysis of 16S V2-V3 rRNA in feces from mice fed acesulfame-K. Band patterns and hierarchical clustering of band patterns of 16S V2-V3 rRNA composition in cecal content (**A**) and feces (**B**) from mice fed acesulfame-K (AK) for 8 weeks, Figure S3: Lumial metabolome analysis in feces from both the control mice in Experiment 1 and 2. Principle component analysis of luminal metabolome data of the control groups from the sucralose experiment (Suc-C) and the acesulfam-K experiment (AK-C), Table S1: Oligonucleotide primer.
