# Supplementary material for: Effects of Low-Dose Non-Caloric Sweetener Consumption on Gut Microbiota in Mice
Source: Nutrients. 2017 Jun 1;9(6):560. doi: 10.3390/nu9060560 (PMC5490539; doi:10.3390/nu9060560)
Supplement: Supplementary file 1 [file nutrients-09-00560-s001.pdf]

**Supplementary Materials**

**Table S1.** Oligonucleotide primer.

| Primer Name | Sequence (5′–3′) | Reference |
|---|---|---|
| Eub338F | ACTCCTACGGGAGGCAGCAG | [33] |
| Eub518R | ATTACCGCGGCTGCTGG | |
| HDA1-GC-F | CGCCCGGGGCGCGCCCCGGGCGGGGCGGGGGCACGGGGGG ACTCCTACGGGAGGCAGCAGT | [34] |
| HDA2-R | GTATTACCGCGGCTGCTGGCAC | |
| Bact934F | GGARCATGTGGTTTAATTCGATGAT | [35] |
| Bact1060R | AGCTGACGACAACCATGCAG | |
| Firm934F | GGAGYATGTGGTTTAATTCGAAGCA | [35] |
| Firm1060R | AGCTGACGACAACCATGCAC | |
| Lactobacillus-F | AGCAGTAGGGAATCTTCCA | [36] |
| Lactobacillus-R | CACCGCTACACATGGAG | |
| Clostridia.IV-F | GCACAAGCAGTGGAGT | [37] |
| Clostridia.IV-R | CTTCCTCCGTTTTGTCAA | |
| ClostridiaXIVa-F | AAATGACGGTACCTGACTAA | [38] |
| Clostridia.XIVa-R | CTTTGAGTTTCATTCTTGCGAA | |
| Bacteroides-F | GAGAGGAAGGTCCCCCAC | [39] |
| Bacteroides-R | CGCTACTTGGCTGGTTCAG | |
| mouse CYP8b1-F | ACAAGCAGCAAGACCTGGAT | NM_010012.3 |
| mouse CYP8b1-R | ATGGAAGAGACGCTGCAACT | |
| mouse CYP7a1-F | AAGTTTCGACATGCTCTCGC | NM_007824.2 |
| mouse CYP7a1-R | CCTCATGGGAGCTGGAATTA | |
| mouse 18s-F | AAACGGCTACCACATCCAAG | NR_003278.3 |
| mouse 18s-R | GGCCTCGAAAGAGTCCTGTA | |

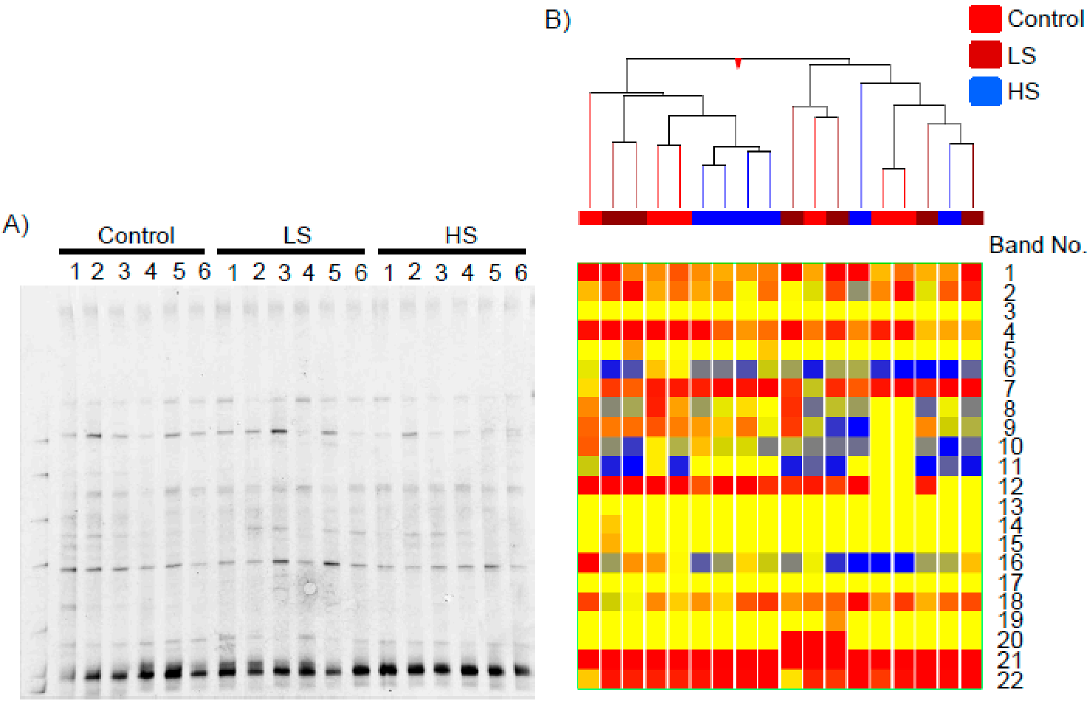


**Figure S1.** DGGE analysis of 16S V2-V3 rRNA in feces from mice fed sucralose. Band patterns (**A**) and hierarchical clustering of band patterns (**B**) for 16S V2-V3 rRNA in feces from mice fed sucralose for 8 weeks. LS: low sucralose (1.5 mg/kg BW/day), HS: high sucralose (15 mg/kg BW/day).

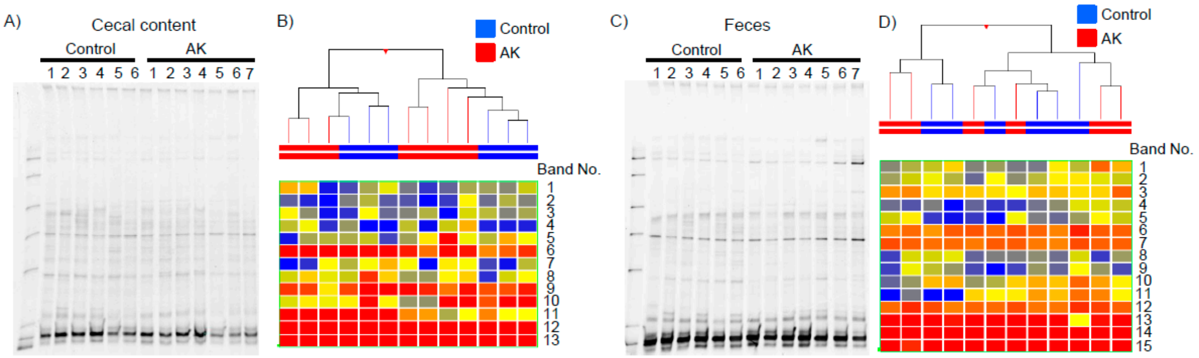


**Figure S2.** DGGE analysis of 16S V2-V3 rRNA in feces from mice fed acesulfame-K. Band patterns and hierarchical clustering of band patterns of 16S V2-V3 rRNA composition in cecal content (**A**) and feces (**B**) from mice fed acesulfame-K (AK) for 8 weeks.

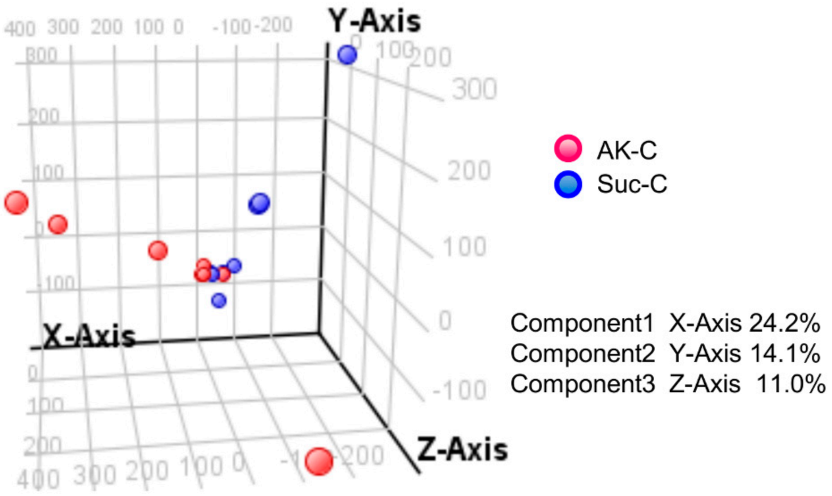


**Figure S3.** Lumial metabolome analysis in feces from both the control mice in Experiment 1 and 2. Principle component analysis of luminal metabolome data of the control groups from the sucralose experiment (Suc-C) and the acesulfam-K experiment (AK-C).
